# Tropism of Highly Pathogenic Avian Influenza H5 Viruses from the 2020/2021 Epizootic in Wild Ducks and Geese

**DOI:** 10.3390/v14020280

**Published:** 2022-01-28

**Authors:** Valentina Caliendo, Lonneke Leijten, Marco van de Bildt, Evelien Germeraad, Ron A. M. Fouchier, Nancy Beerens, Thijs Kuiken

**Affiliations:** 1 Department of Viroscience, Erasmus Medical Center, 3015 GD Rotterdam, The Netherlands; l.leijten@erasmusmc.nl (L.L.); m.vandebildt@erasmusmc.nl (M.v.d.B.); r.fouchier@erasmusmc.nl (R.A.M.F.); t.kuiken@erasmusmc.nl (T.K.); 2 Department of Virology, Wageningen Bioveterinary Research, 8221 RA Lelystad, The Netherlands; evelien.germeraad@wur.nl (E.G.); nancy.beerens@wur.nl (N.B.)

**Keywords:** avian influenza, HPAI, H5N8, Eurasian wigeon, barnacle goose, enterotropism, neurotropism

## Abstract

Highly pathogenic avian influenza (HPAI) outbreaks have become increasingly frequent in wild bird populations and have caused mass mortality in many wild bird species. The 2020/2021 epizootic was the largest and most deadly ever reported in Europe, and many new bird species tested positive for HPAI virus for the first time. This study investigated the tropism of HPAI virus in wild birds. We tested the pattern of virus attachment of 2020 H5N8 virus to intestinal and respiratory tissues of key bird species; and characterized pathology of naturally infected Eurasian wigeons (*Mareca penelope*) and barnacle geese (*Branta leucopsis*). This study determined that 2020 H5N8 virus had a high level of attachment to the intestinal epithelium (enterotropism) of dabbling ducks and geese and retained attachment to airway epithelium (respirotropism). Natural HPAI 2020 H5 virus infection in Eurasian wigeons and barnacle geese also showed a high level of neurotropism, as both species presented with brain lesions that co-localized with virus antigen expression. We concluded that the combination of respirotropism, neurotropism, and possibly enterotropism, contributed to the successful adaptation of 2020/2021 HPAI H5 viruses to wild waterbird populations.

## 1. Introduction

Highly pathogenic avian influenza (HPAI) outbreaks have become increasingly frequent in wild bird populations and have caused mass mortality in many wild bird species [1,2,3,4,5,6,7,8,9]. Recently circulating HPAI H5 viruses descending from the A/Goose/Guangdong/1/1996 (GsGd) lineage, principally HPAI H5N8 and H5N1 viruses belonging to clade 2.3.4.4, were responsible for the latest outbreak events [6,7,8,9].

In Europe, outbreaks coincide with the autumn migrations of wild birds, a time when many long-distance migratory waterbirds congregate to overwinter or stop-over around water-rich areas [10]. The two most deadly HPAI epizootics occurred in Europe during the avian influenza seasons in 2016/2017 and in 2020/2021 [11,12]. In the Netherlands, over 13,000 wild birds were reported dead during the 2016/2017 epizootic; duck species such as Eurasian wigeons (*Mareca penelope*) and tufted ducks (*Aythya fuligula*) accounted for the highest rates of mortality [11]. The 2020/2021 epizootic was the largest and most deadly ever reported in Europe; geese, in particular barnacle goose (*Branta leucopsis*) and graylag goose (*Anser anser*), experienced among the highest rates of mortality [6,7,8,9]. Furthermore, like the above-mentioned barnacle goose, several new bird species were reported infected with HPAI virus for the first time. Temporally, the virus continued to circulate within Europe well after the periods of autumn migration and wintering, and it was regularly detected in wild birds during the following 2021 summer [9]. During the first months of the 2020/2021 epizootic, H5N8 was the most frequently detected virus subtype; however, this virus subtype locally reassorted with low pathogenic avian influenza viruses (LPAIVs), so that many different virus subtypes (i.e., H5N1, H5N2, H5N3, H5N4, H5N5, H5N8) were subsequently identified [6,7,8,9,13].

Both experimental studies and field observations identified wild ducks, and in particular dabbling species such as Eurasian wigeons and mallards, as the most suitable vectors for the long-distance spread of HPAI viruses [1,2,14,15]. Wild ducks can be infected with HPAI virus without presenting any clinical signs [15]. During the 2020/2021 epizootic, only a relatively small number of infected Eurasian wigeons and mallards (*Anas platyrhynchos*) were reported dead in Europe during surveillance [6,7,8,9]. In some cases, birds of these species tested positive after being professionally hunted and were reported as otherwise apparently healthy. These findings indicate a relatively lower virulence of H5 viruses in some duck species and may support the theory of increased adaptation and replication of HPAI virus in these species. Viral adaptation may relate to an increased viral enterotropism in these hosts, as is found for low pathogenic avian influenza viruses that are enzootic in some waterbird species. A previous study already showed the increased enterotropism of 2016 H5N8 [16].

In this study, we investigated whether 2020 H5N8 virus followed the trend of its predecessor, 2016 H5N8 virus, of increased enterotropic potential. We hypothesized that enterotropism is likely to be maintained, even enhanced, especially in bird species such as the mallard and Eurasian wigeon that are hypothesized as being vectors for long-distance viral dispersion. Furthermore, enterotropism could be also evident in new host species, such as graylag goose and barnacle goose, with the advantage of virus perpetuation in these globally abundant host populations and their movements.

Our study is based on the hypothesis that the 2020 HPAI H5N8 virus was more enterotropic than the 2016 HPAI H5N8 virus, both in established and in new host species. To investigate this hypothesis, we determined the pattern of attachment of the 2020 H5N8 virus to intestinal and respiratory tissues of key species of wild ducks and geese, as well as chickens, and then compared it with the patterns of attachment of older HPAI viruses such as H5N1, 2014 H5N8, and 2016 H5N8 viruses. Furthermore, we examined wild Eurasian wigeons and barnacle geese that died during the 2020/2021 epizootic and determined virus distribution, tissue tropism and associated lesions in their major organs. Because the barnacle goose is a new host species for HPAI virus, associated pathological findings in this species have not yet been described. Because HPAI virus-infected geese have been reported to show mainly neurological clinical signs (e.g., tremors, incoordination, torticollis) [7], we were particularly interested in investigating whether the virus was neurotropic in these birds.

## 2. Materials and Methods

### 2.1. Study Design

Our study consisted of two parts. First, we performed virus histochemical analysis of four HPAI viruses to compare the pattern of attachment of these viruses in the respiratory and intestinal tracts of five bird species. We were particularly interested to determine whether 2020 H5N8 virus attached similarly to the 2016 H5N8, 2014 HPAI H5N8 and 2005 H5N1 viruses to the digestive tract of three wild duck species (Eurasian wigeon; mallard; tufted duck) and one key wild goose species (graylag goose). Second, we examined the carcasses of 14 wild birds (5 Eurasian wigeons and 9 barnacle geese) that were found dead during the 2020/2021 H5N8 HPAI epidemic in the Netherlands and that tested positive for H5 HPAI virus, to characterize the virology, pathology, and cell type tropism of this virus infection in different organs. The Eurasian wigeons were freshly dead at the time of necropsy; the barnacle geese were at different stages of autolysis, ranging from freshly dead to mildly autolyzed. We were particularly interested to determine whether 2020 H5N8 HPAI virus had similar tropism as 2016 H5N8 HPAI virus, from the 2016/17 epidemic, for the digestive tract of wild birds. The techniques of investigation were similar to Caliendo et al., 2020 [16], and they are described below.

### 2.2. Virus Histochemistry

The following four virus isolates were used: 2005 HPAI virus H5N1 (A/Turkey/Turkey/1/05), 2014 HPAIV H5N8 (A/Eurasian wigeon/Netherlands/emc-1/2014), 2016 HPAI virus H5N8 (A/Eurasian wigeon/Netherlands/19/2016), and 2020 HPAI virus H5N8 (A/Eurasian Wigeon/Netherlands/3/2020). The viruses were individually passaged in Madin-Darby canine kidney (MDCK) cells. After 2–3 days, the supernatant was harvested and cleared of cell debris by low-speed centrifugation for 20 min at 1455× *g*. The viruses were individually concentrated by centrifugation of the cleared supernatants in filter tubes (Amicon Ultra-15 100K filter-tubes, Millipore, UFC9100024, Darmstadt, Germany) for 40 min at 4000× *g* at 4 °C. The concentrated virus was inactivated by dialysing against 0.1% formalin for 3 days at room temperature (RT). After inactivation, the virus solution was dialysed against phosphate-buffered saline solution (PBS) and complete inactivation was confirmed by passaging twice on MDCK cells. Virus was labelled by adding an equal volume of 0.1 mg/mL of fluoresceinisothiocyanate (FITC) (Sigma-Aldrich, Saint Louis, MO, USA) in 0.5 M bicarbonate buffer (pH 9.5) for 1 h at RT while constantly stirring. Labelled virus was dialysed against PBS in order to lose all unbound FITC. The concentration of the different virus suspensions used for virus histochemistry was standardized at 50 hemagglutination units/100 μL (HAU) using hemagglutination assay with turkey red blood cells.

Tissue sections of the following species were used: tufted duck (*n* = 3), Eurasian wigeon (*n* = 3), mallard (*n* = 3), graylag goose (*n* = 3) and chicken (Gallus domesticus) (*n* = 2). These tissues came from the Erasmus MC tissue bank, and were from healthy animals that showed no abnormalities or histological lesions. From the digestive tract of the same birds, tissues selected were duodenum, jejunum, ileum and colon. Sections of jejunum and ileum of graylag goose were not available for virus histochemistry because of limitations of sampling due to high workload during HPAI outbreak. From the respiratory tract, tissues selected were trachea, primary bronchus, secondary bronchus, tertiary bronchus or parabronchus, air capillaries and air sacs.

Three-μm-thick formalin-fixed paraffin-embedded sections of each tissue were deparaffinized in xylene and hydrated using graded alcohols and incubated overnight at 4 °C with FITC-labelled viruses at a concentration of 50 HAU/100 μL. To enable visualization by light microscopy, FITC was linked to a peroxidase-labeled rabbit anti-FITC antibody (DAKO, Glostrup, Denmark). The signal was amplified using a tyramide amplification system (Perkin-Elmer, Boston, MA, USA). Peroxidase was revealed with 3-amino-9-ethylcarbazole (Sigma-Aldrich) resulting in a bright red precipitate. Tissues were counterstained with hematoxylin and embedded in glycerol-gelatin (Merck, Whitehouse Station, NJ, USA). Omission of the FITC-labelled virus was used as a negative control.

The slides were assessed with light microscopy to estimate the abundance of viral attachment to epithelial cells and scored as follows: attachment to <1% of epithelial cells (−), attachment to ≥1 and <10% of epithelial cells (±), attachment to ≥10% and <50% of epithelial cells (+), and attachment to ≥50% of epithelial cells (++). Finally, the median score was determined for each species at the different anatomical sites. Sections were examined without knowledge of the identity of the birds.

### 2.3. Virology, Pathology and Immunohistochemistry of Naturally Infected Wild Birds

The carcasses of 14 wild birds (5 Eurasian wigeons and 9 barnacle geese) had been collected in the Netherlands in November and December 2020. Pharyngeal and cloacal swabs were collected from each bird for virus diagnostics, using sterile cotton swabs placed in 1 mL of virus transport medium. All the birds tested positive for HPAIV H5 2020 by real-time reverse-transcription PCR (RRT-PCR) assays in oropharyngeal and/or cloacal swabs as described previously [12].

At postmortem examination, the following tissues, when available, were examined: brain, lungs, air sacs, pancreas, liver, stomachs (proventriculus and ventriculus), small intestine (duodenum, jejunum at 5, 15, 35 cm along its length, ileum, ileocaecal junction), large intestine (cecum, colon), kidney, adrenal gland, spleen and heart. Duplicate tissue sections were collected for virus detection and kept at −80 °C until analysis, or were fixed in 10% neutral-buffered formalin and embedded in paraffin. Organs of barnacle geese were not available for virology. For virus detection, tissue samples were first weighed and then homogenized with a FastPrep 24 (MP Biomedicals, Eindhoven, The Netherlands) in Hank’s balanced salt solution and centrifuged briefly before dilution in lysis buffer for RNA extraction [14,15]. Extracted total RNA of tissue samples, pharyngeal and cloacal swabs were tested for the presence of influenza A virus matrix gene-fragment; viruses in pharyngeal and cloacal swabs were further subtyped using real-time RT-PCR targeting fragments of the H5 and N8 genes [17]. Briefly, RNA from the tissue and swab suspensions was isolated using a MagNaPure LC System with the MagNaPure LC Total Nucleic Acid Isolation Kit (Roche Diagnostics, Almere, The Netherlands). RT-PCR were performed on an ABI 7700 machine (Applied Biosystems, Foster City, CA, USA) using a TaqMan Fast Virus 1-Step Master Mix (Applied Biosystems). The oligonucleotides (5′-CTT-CTR-ACC-GAG-GTC-GAA-ACG-TA-3′) and (5′-TCT-TGT-CTT-TAG-CCA-YTC-CAT-GAG-3′) and the labeled probes (5′ 6-FAM-TCA-GGC-CCC-CTC-AAA-GCC-GAG-A-BHQ-3′) and (5′ 6-FAM-TCA-GGC-CCC-CTC-AAA-GCC-GAA-A-BHQ-3′) were used for detection of the matrix segment of influenza A viruses. Samples were considered virus-positive if the cycle threshold (Ct) value was <40 [14,15,17]. For histopathology and immunohistochemistry, tissues were sectioned at 3 µm and stained with hematoxylin and eosin for histopathological analysis or stained with a monoclonal antibody against nucleoprotein of influenza A virus for immunohistochemical detection of influenza viral antigen, as described previously [14,15].

## 3. Results

### 3.1. Pattern of Virus Attachment to Epithelia of Digestive and Respiratory Tracts

In intestinal epithelia, 2014 H5N8, 2016 H5N8, and 2020 H5N8 had overall higher attachment than H5N1 (Table 1).

In comparison among host species, virus attachment to intestinal epithelia was low in tufted duck, intermediate in Eurasian wigeon and graylag goose, and high in chicken, mallard. The level of attachment of 2020 H5N8 per host species was: in mallard, similar to 2016 H5N8 and higher than H5N1 and 2014 H5N8; in Eurasian wigeon, similar to 2014 H5N8, higher than H5N1 and slightly higher than 2016 H5N8; in tufted duck lower than 2014 H4N8, 2016 H5N8 and H5N1; in graylag goose, slightly higher than 2014 H5N8, 2016 H5N8 and H5N1, because of the higher attachment to the duodenum; in chicken, all the viruses had the same, high level of attachment.

In respiratory epithelia, the virus attachment of each virus was generally high in all species, especially for trachea, primary and secondary bronchus (Table 2).

The level of attachment of 2020 H5N8 was lower than 2016 H5N8 in secondary bronchi of tufted duck and air capillaries of graylag goose; and higher than 2016 H5N8 in the air capillaries of chicken.

### 3.2. Virology, Influenza Virus Antigen Expression and Associated Lesions in Naturally Infected Wild Birds

For Eurasian wigeons, pharyngeal and cloacal swabs were positive in all five tested birds (Table 3).

Viral RNA was detected in all the organs tested (i.e., lung, liver, heart, jejunum, brain), meaning that the virus had spread systemically.

For barnacle geese, cloacal swabs were more frequently positive than pharyngeal swabs (88% cloacal swab, positive/tested 8/9; 66% pharyngeal swab, positive/tested 6/9), but, when both positive, they overall contained a similar amount of virus.

**Figure 1:**
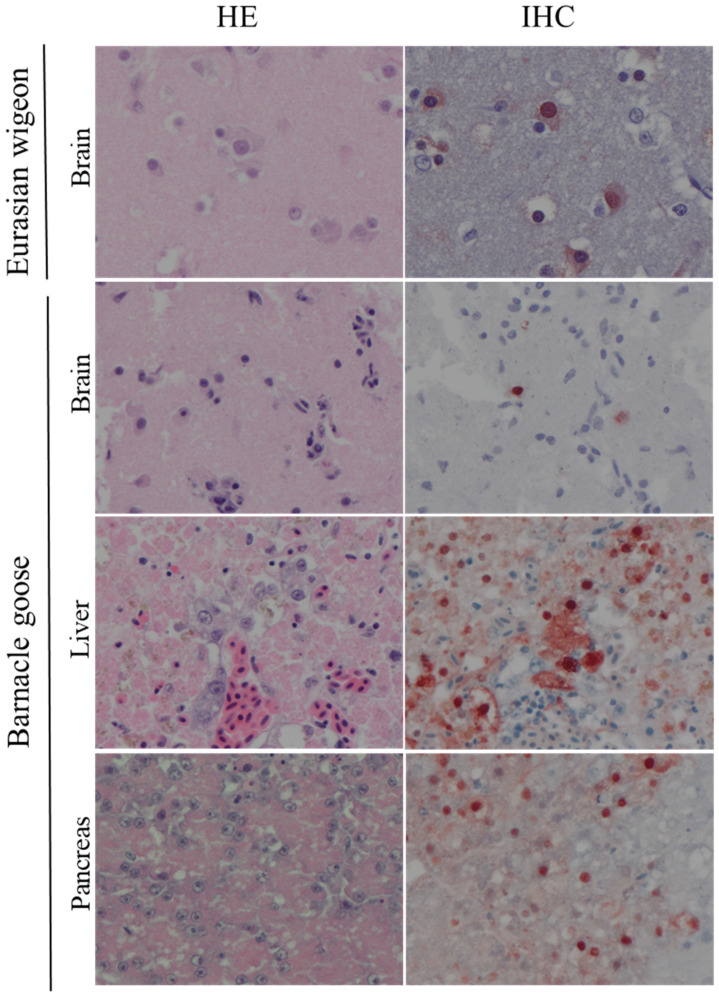
Histological changes and influenza virus antigen expression in tissues of H5-infected Eurasian wigeons (*Mareca penelope*) and Barnacle geese (*Branta leucopsis*). Tissue sections on the left column are stained with hematoxylin and eosin (HE). Serial tissue sections in the right column are stained for influenza virus antigen by immunohistochemistry (IHC). In all tissues there is necrosis and inflammation associated with virus antigen expression.

Grossly, the main pathological changes consisted of multifocal necrosis in liver and pancreas; pin-point hemorrhages in the brain; sub-pericardial hemorrhages; and multifocal pulmonary. Histologically, lesions were detected in the liver, brain, kidney, lung, heart (Figure 1, Table 4 and Table 5).

In liver and pancreas, lesions were characterized as multifocal, mild-to-moderate necrosis; these necrotic foci co-localized with presence of viral antigen. In the brain, lesions were characterized as multifocal encephalitis with foci of gliosis, neuronal degeneration, and necrosis; abundant viral antigen was present in the nucleus and cytoplasm of several neurons in these lesions. In kidney, lesions were characterized as mild interstitial nephritis, with no presence of viral antigen. In the heart, lesions were characterized as multifocal to focally extensive myocardial necrosis; few myocardial cells in these lesions presented viral antigen. Mild-to-moderate inflammatory changes and infiltration with mononuclear cells were observed in lung, and few epithelial cells in these lesions presented viral antigen. There were no histological lesions and no detectable viral antigen in the gastro-intestinal tract of the examined birds.

## 4. Discussion

The high number of birds and the increasing number of wild bird species infected during recent HPAI outbreaks suggest that HPAI H5 viruses are adapting to wild birds. During past epizootics, domestic birds had played an important role for the Gs/Gd lineage by acting as a main reservoir where virus evolution could take place [1,2]. Gradually, the virus adapted better to wild birds and now appears to be maintained in wild populations independently of domestic bird populations [6,7,8,9]. One of the factors behind this adaptation may be the newly acquired tropism of HPAI viruses for the intestinal tract of their hosts [16]. Enterotropism is a mechanism more commonly seen for LPAIVs, but also recently reported for HPAI H5N8, and allow fecal-oral virus transmission in wild birds [18]. The findings of this study partially support our hypothesis that 2020 H5N8 virus has higher tropism for the intestinal tract of wild birds than 2016 H5N8 virus. First, 2020 H5N8 virus had slightly higher level of attachment to the intestinal epithelium of Eurasian wigeon and graylag goose. In mallards, the level of attachment for the intestinal tract of the 2020 H5N8 virus was unchangingly high compared to the 2016 H5N8 virus. The tufted duck is a less likely long-distance vector for HPAIV, and the 2020 H5N8 virus had a low level of attachment to the intestinal epithelium of this species.

Second, cloacal swabs were consistently positive for viral RNA in naturally infected Eurasian wigeons and barnacle geese, and were more reliable in detecting the infection in barnacle geese. In addition, the jejunum of the five infected Eurasian wigeons was consistently positive for virus RNA by RT-PCR. These findings contrast with previous studies, where the cloacal excretion of HPAI virus infected birds was usually uncommon and, if present, lower than pharyngeal excretion [14,15,16]. These findings support the possibility of HPAI virus replication in the intestinal tract of wild birds.

However, a finding against our hypothesis was the lack of virus antigen expression and associated histological lesions in the intestinal epithelium of naturally infected Eurasian wigeons and barnacle geese. Virus antigen expression had been previously reported in the gastro-intestinal epithelium of Eurasian wigeon and graylag goose naturally infected with 2016 H4N8 [16]. Therefore, we could not confirm 2020 H5N8 virus replication in the intestinal tract of these birds.

Eurasian wigeons and geese have similar biological traits that may be relevant for the epidemiology of HPAI. Eurasian wigeons have already been described as long-distant vector; HPAI virus infection in barnacle and graylag geese is instead a more recent and less-studied event. Although migratory goose species were not previously found to disperse LPAIVs [19], this question should be revisited for HPAI viruses given the novel involvement of barnacle geese and graylag geese in recent HPAI epizootics.

Another common trait between Eurasian wigeons and geese is their feeding biology. Apart from dabbling, Eurasian wigeons use a diverse feeding strategy that includes foraging by grazing on land [20,21]. During migration, wigeons make use of a great variety of wetland habitats, including wet farm fields, and often feed on grass, leaves, stems and roots. The use of water-rich land for resting and feeding is also a common feature in geese, as these birds regularly congregate and feed on improved agricultural pastures [22,23]. During these events, a high density of birds makes use of limited surface space. The close contact, and the fact that birds contaminate the grass that they eat with potentially infected feces, may increase chances of AIV infection via the orofecal route in flocks of Eurasian wigeons as well as geese.

Respirotropism is retained for the 2020 H5N8 virus, based on patterns of virus attachment and virus antigen expression observed in this study. The combination of these two mechanisms (i.e., respirotropism and enterotropism) could give HPAI viruses an advantage over LPAIVs that are mainly excreted via the orofecal route.

This study also described the virology and pathology of HPAI virus infection in barnacle geese, a new host species for the virus. Infection with HPAI H5 viruses in both Eurasian wigeons and barnacle geese was characterized by a high level of neurotropism. Both species presented with brain lesions associated with virus antigen expression. Most likely, this also represented the cause of death for the birds. Brain lesions were also compatible with neurological signs (i.e., incoordination, body tremors, torticollis) shown by many birds and described by field operators during the 2020/2021 HPAI surveillance [7]. The high neurotropic potential of HPAI viruses has been reported in domestic species, and it is more often reported also in wild birds [24,25,26,27]. In previous studies, multifocal viral encephalitis associated with severe neurological signs were observed in tufted ducks and common pochards (*Aythya ferina*) infected with HPAI 2005 H5N1 virus [14]; polioencephalitis, and associated high viral loads in the brain, were observed in Pekin ducks (*Anas platyrhynchos domesticus*) infected with HPAI 2016 H5N8 virus [27]. Similarly, pronounced neurotropism characterized by multifocal encephalitis with foci of gliosis, neuronal degeneration and necrosis was observed in Eurasian wigeons and barnacle geese infected with HPAI 2020 H5 viruses.

The mortality of waterbirds from the neurotropism of the 2020 HPAIV H5N8 could serve in reaching yet more new host species. Infected waterbirds with neurological signs are more visible and trigger a hunt response of their predators; they are easier prey to catch and more likely to be eaten [28,29]. In 2020/2021, newly HPAI virus infected species were predatory birds such as great skua (*Stercorarius skua*) and golden eagle (*Aquila chrysaetos*) [8,9]. In addition, for the first time since the HPAI H5N1 epidemic in 2005/2006, during the 2020/2021 HPAI H5 epidemic wild mammalian carnivores were reported to be HPAI virus-infected; European foxes (*Vulpes vulpes*), gray seals (*Halichoerus grypus*) and harbor seals (*Phoca vitulina*) were found dead, probably from contact or ingestion of infected wild birds [8,9]. Because of the relatively limited history of HPAI in wild birds, it is possible that virulence levels have not yet been optimized for transmissibility by natural selection [30,31], and the continuously evolving dynamics of HPAI in wild birds may bring new directions for virulence and tropism of HPAI virus in wild birds.

In conclusion, HPAI H5 viruses from the 2020/2021 epidemic expressed a diverse repertoire of tissue tropism, including respirotropism and neurotropism, as well as a high level of in vitro enterotropism, in infecting wild ducks and geese. Because of the constant evolution shown by HPAI viruses, continued monitoring of the future outbreaks is vital to better understand the new dynamics between virus and its hosts.

## Figures and Tables

**Table 1:** Pattern of attachment of avian influenza viruses to the epithelial cells of the intestinal tract.

		Avian Influenza Viruses			
Species	Tissues	2005 H5N1	2014 H5N8	2016 H5N8	2020 H5N8
Mallard	Duodenum	++	+	++	++
	Jejunum	++	++	++	++
	Ileum	+	+	++	++
	Colon	+	+	++	++
Eurasian wigeon	Duodenum	±	±	±	±
	Jejunum	−	±	±	±
	Ileum	−	±	−	±
	Colon	−	±	−	±
Tufted duck	Duodenum	±	±	±	±
	Jejunum	−	±	±	−
	Ileum	−	±	−	−
	Colon	±	±	−	−
Graylag goose	Duodenum	±	±	±	+
	Jejunum	nd	nd	nd	nd
	Ileum	nd	nd	nd	nd
	Colon	±	±	±	±
Chicken	Duodenum	++	++	++	++
	Jejunum	++	++	++	++
	Ileum	++	++	++	++
	Colon	+	+	+	+

Mean abundance of attachment was scored as follows: attachment to <1% of epithelial cells (−), attachment to ≥1 and <10% of epithelial cells (±), attachment to ≥10% and <50% of epithelial cells (+), and attachment to ≥50% of epithelial cells (++). nd, not done.

**Table 2:** Pattern of attachment of avian influenza viruses to the epithelial cells of the respiratory tract.

		Avian Influenza Viruses			
Species	Tissues	2005 H5N1	2014 H5N8	2016 H5N8	2020 H5N8
Mallard	Trachea	++	++	++	++
	Primary bronchus	++	++	++	++
	Secondary bronchus	++	++	++	++
	Parabronchus atria	±	±	±	±
	Air capillaries	+	±	±	±
	Air sac	++	++	++	++
Eurasian wigeon	Trachea	+	++	++	++
	Primary bronchus	+	++	++	++
	Secondary bronchus	++	++	++	++
	Parabronchus atria	±	±	±	±
	Air capillaries	+	±	±	±
	Air sac	++	++	++	++
Tufted duck	Trachea	++	+	++	++
	Primary bronchus	++	++	++	++
	Secondary bronchus	++	++	++	±
	Parabronchus atria	+	±	±	±
	Air capillaries	+	±	±	±
	Air sac	++	+	++	++
Graylag goose	Trachea	++	++	++	++
	Primary bronchus	nd	nd	nd	nd
	Secondary bronchus	++	++	++	++
	Parabronchus atria	++	+	+	+
	Air capillaries	+	+	++	+
	Air sac	++	++	++	++
Chicken	Trachea	++	++	++	++
	Primary bronchus	+	++	++	++
	Secondary bronchus	+	++	++	++
	Parabronchus atria	++	+	++	++
	Air capillaries	+	−	±	+
	Air sac	++	++	++	++

Mean abundance of attachment was scored as follows: attachment to <1% of epithelial cells (−), attachment to ≥1 and <10% of epithelial cells (±), attachment to ≥10% and <50% of epithelial cells (+), and attachment to ≥50% of epithelial cells (++). nd, not done.

**Table 3:** RT-PCR results * in swabs and organs of H5N8-infected wild Eurasian wigeons (*Mareca penelope*) and barnacle goose (*Branta leucopsis*).

Samples	Eurasian Wigeons					Barnacle Geese								
	W1	W2	W3	W4	W5	G1	G2	G3	G4	G5	G6	G7	G8	G9
Virus	H5N8	H5N1	H5N8	H5N8	H5N8	H5N8	H5N8	H5N8	H5N8	H5N8	H5N8	H5	H5N8	H5N8
Pharyngeal swab	22	30	28	33	23	np	27	29	33	np	33	np	22	27
Cloacal swab	30	27	26	24	27	28	29	np	28	30	29	34	28	28
Lung	25	32	23	31	27	nd	nd	nd	nd	nd	nd	nd	nd	nd
Liver	29	30	35	34	22	nd	nd	nd	nd	nd	nd	nd	nd	nd
Heart	23	30	23	32	27	nd	nd	nd	nd	nd	nd	nd	nd	nd
Jejunum	27	30	23	30	22	nd	nd	nd	nd	nd	nd	nd	nd	nd
Brain	28	34	24	32	28	nd	nd	nd	nd	nd	nd	nd	nd	nd

* Cycle threshold (Ct) value, cut-off value is 40; np, not present; nd, not done.

**Table 4:** Frequency and distribution of gross and histological lesions in carcasses of wild birds.

Number of Birds with Gross (G) and Histological (H) Lesions in the:																	
		Respiratory System				Digestive System						Other Systems					
		Lung		Air Sac		Intestine		Pancreas		Liver		Brain *		Heart		Kidney	
Species	No of Birds	G	H	G	H	G	H	G	H	G	H	G	H	G	H	G	H
Eurasian wigeon	5	3	0	0	0	0	0	1	0	0	1	1	3	1	1	0	0
Barnacle goose	9 *	3	2	0	0	0	0	5	3	0	1	3	4	2	0	0	1

* available for 4 birds.

**Table 5:** Expression of avian influenza virus antigen in cell types of different organs of wild birds.

Number of Birds Expressing Influenza Virus Antigen in a Cell Type of an Organ																		
		Respiratory System						Digestive System							Other Systems			
		Lung			Air Sac			Intestine			Pancreas		Liver		Brain *		Heart	
Species	No of Birds	EP	E	N	EP	E	N	EP	E	N	E	EP	E	H	E	N	E	M
Eurasian wigeon	5	0	0	0	0	0	0	0	0	0	0	0	0	0	1	3	0	1
Barnacle goose	9 *	2	0	0	0	0	0	0	0	0	0	2	0	1	0	4	0	0

Ep, epithelial cell; E, endothelial cell; N, neuron; H, hepatocyte; M, myocytes; * available for 4 birds.

## Data Availability

All data are available in the main text.
